# S100A9 in adult asthmatic patients: a biomarker for neutrophilic asthma

**DOI:** 10.1038/s12276-021-00652-5

**Published:** 2021-07-20

**Authors:** Quang Luu Quoc, Youngwoo Choi, Tra Cao Thi Bich, Eun-Mi Yang, Yoo Seob Shin, Hae-Sim Park

**Affiliations:** 1 Department of Allergy and Clinical Immunology, Ajou University School of Medicine, Suwon, South Korea; 2 Department of Biomedical Sciences, Ajou University School of Medicine, Suwon, South Korea

**Keywords:** Prognostic markers, Adaptive immunity, Immunological disorders, Diagnostic markers

## Abstract

The biomarkers and therapeutic targets of neutrophilic asthma (NA) are poorly understood. Although S100 calcium-binding protein A9 (S100A9) has been shown to correlate with neutrophil activation, its role in asthma pathogenesis has not been clarified. This study investigated the mechanism by which S100A9 is involved in neutrophil activation, neutrophil extracellular trap (NET)-induced airway inflammation, and macrophage polarization in NA. The S100A9 levels (by ELISA) in sera/culture supernatant of peripheral blood neutrophils (PBNs) and M0 macrophages from asthmatic patients were measured and compared to those of healthy controls (HCs). The function of S100A9 was evaluated using airway epithelial cells (AECs) and PBNs/M0 macrophages from asthmatic patients, as well as a mouse asthma model. The serum levels of S100A9 were higher in NA patients than in non-NA patients, and there was a positive correlation between serum S100A9 levels and sputum neutrophil counts (*r* = 0.340, *P* = 0.005). Asthmatic patients with higher S100A9 levels had lower PC_20_ methacholine values and a higher prevalence of severe asthma (SA) (*P* < .050). PBNs/M0 macrophages from SA released more S100A9 than those from non-SA patients. PBNs from asthmatic patients induced S100A9 production by AECs, which further activated AECs via the extracellular signal-regulated kinase (ERK) pathway, stimulated NET formation, and induced M1 macrophage polarization. Higher S100A9 levels in sera, bronchoalveolar lavage fluid, and lung tissues were observed in the mouse model of NA but not in the other mouse models. These results suggest that S100A9 is a potential serum biomarker and therapeutic target for NA.

## S100A9 as a biomarker for neutorphilic asthma

A calcium-binding protein called S100A9 could be used as a biomarker for diagnosing a phenotype of asthma called neutrophilic asthma, and might also be a suitable target for drugs to treat the condition. Hae-Sim Park and colleagues at Ajou University in Suwon, South Korea, investigated the mechanism by which S100A9 activates neutrophils to induce the airway inflammation and other changes associated with asthma. Patients with neutrophilic asthma had higher levels of the protein in their blood. Measuring these levels could therefore be used as a potential biomarker. Serum biomarkers for identifying neutrophilic asthma are currently lacking. The research also supports the hypothesis that S100A9 plays a critical role in initiating and perpetuating the neutrophilic asthma and could become the therapeutic target.

## Introduction

Asthma is a heterogeneous chronic inflammatory lung disease characterized by airway inflammation and remodeling^1^. The prevalence, severity, and mortality of asthma are considered globally because this disease affects more than 350 million people worldwide^2^. Severe asthma (SA) (5–10% of all asthmatic patients) is a distinct phenotype of asthma in individuals suffering from frequent asthma exacerbations and progressive lung function decline even with a higher dose of medication, having persistent eosinophilia, as well as neutrophilia^3–6^. A recent study classified asthma into 4 phenotypes (eosinophilic [EA], neutrophilic [NA], mixed granulocytic [MA], and pauci-granulocytic types) according to sputum inflammatory cell profiles^7^. NA was defined as 65% or more neutrophils among the total cell number^8^. Patients with NA are characterized by steroid resistance, frequent asthma exacerbations, and persistent or severe symptoms^5,8^. Unlike those of EA, there are still a large number of unmet needs for NA: (1) a lack of suitable biomarkers for categorizing this phenotype and predicting the response to inhaled corticosteroids, (2) a lack of biomarkers to assess the extent of NA, as blood neutrophil counts do not reflect airway neutrophilia, and (3) currently available biologics for SA focusing on type 2 asthma but not NA or non-type 2 asthma^9^.

The close interactions between neutrophils and airway epithelial cells (AECs), macrophages, and T helper (Th) 17 cells have been demonstrated to play integral roles in the pathogenesis of neutrophilic airway inflammation through the release of cytokines^10^. In particular, interleukin (IL)-17 release by Th17 cells due to the loss of the Th17-Treg cell balance may increase CXC chemokine release and neutrophilia in the airway in a murine model of asthma^11,12^. Moreover, IL-8 and tumor necrosis factor α (TNF-α) are released mainly by macrophages and have been suggested to be major inflammatory mediators related to the development of airway hyperresponsiveness (AHR) in patients with NA^12,13^. S100 Calcium-Binding Protein A9 (S100A9), also known as migration inhibitory factor-related protein 14, is secreted by activated neutrophils, macrophages, and AECs^14^. Higher levels of S100A9 were observed in the sera and sputa of patients with NA, especially in those with uncontrolled asthma^15–17^. Additionally, S100A9 is involved in innate immune responses (with activation of AECs and macrophages) under the regulation of Toll-like receptor 4 during the pathogenesis of Baker’s asthma^18^. Therefore, we hypothesized that S100A9 may be a key mediator that regulates airway inflammation, remodeling, and immune cell activation in NA to contribute to the poor clinical outcomes of NA.

The present study investigated the role of S100A9 in NA by (1) evaluating whether S100A9 was a potential biomarker to discriminate NA from non-NA, (2) elucidating the mechanism by which S100A9 enhances neutrophil activation, neutrophil extracellular DNA trap (NET) formation, and macrophage polarization in vitro, in vivo, and ex vivo, and (3) evaluating the effect of anti-S100A9 antibodies on these inflammatory pathways.

## Materials and methods

### Study subjects

Asthmatic patients were recruited at the Department of Allergy and Clinical Immunology at Ajou University Hospital, Suwon, South Korea. They were composed of 187 adult asthmatic patients who were taking anti-inflammatory medications, including inhaled corticosteroids, for more than 2 years and 57 healthy controls (HCs). No one had maintained systemic steroids. Asthma diagnosis was based on recurrent episodes of wheezing, dyspnea, cough, and sputum production, as well as either AHR to methacholine (MCh) or reversible airway obstruction improved by a short‐acting β2-agonist according to the GINA guideline^19^. Atopic status was defined by skin prick test results as previously described^20^. Patient serum was collected at the initial visit. Serum total IgE was measured using the ImmunoCAP system (ThermoFisher Scientific, Waltham, CA, USA). SA was defined according to the International European Respiratory Society/American Thoracic Society Guidelines^4^. The NA phenotype was defined as having >65% sputum neutrophils^21,22^. Lung function FEV_1_% (forced expiratory volume in 1 s, % predicted values) was evaluated using spirometry, and MCh challenge tests were performed as previously described^20^. Serum samples were collected and stored at −70 °C for further analysis. This study was approved by the Institutional Review Board of Ajou University Hospital (AJIRB‐BMR‐SUR‐15‐498), and all the subjects provided written informed consent.

### Epithelial cell culture and treatment protocols

The human lung carcinoma cell line (A549) and primary small airway epithelial cells (SAECs) were obtained from the American Type Culture Collection (ATCC) (Manassas, VA, USA). A549 cells were cultured in RPMI‐1640 medium supplemented with 10% fetal bovine serum, 100 U/mL penicillin G sodium, and 100 μg/mL streptomycin sulfate (all from Gibco, Grand Island, NY, USA) at 37 °C with 5% CO_2_ in humidified air. SAECs were cultured to passages 3‐8 in AEC-basal medium supplemented with the Bronchial Epithelial Cell Growth Kit (all from the ATCC). A549 (2 × 10^5^ cells) and SAECs (1×10^5^ cells) were treated with 100 ng/mL lipopolysaccharide (LPS) (Sigma‐Aldrich, St. Louis, MO, USA) or S100A9 (Abcam, Cambridge, MA, USA) for 24 h. Supernatants were harvested for further ELISA analysis. For western blot analysis, AECs were treated with S100A9 for 24, 48, or 72 h to evaluate the expression of tight junction proteins and airway remodeling-related signaling and incubated for the indicated times (0, 5, 15, 30, 60, and 120 min) to examine the expression of p38, extracellular signal-regulated kinase (ERK), and nuclear factor kappa B (NF-κB) pathway factors. In some experiments, A549 cells and SAECs were cocultured with peripheral blood eosinophils (PBEs) (ratio 1:5), peripheral blood neutrophils (PBNs) (ratio 1:5), or M1 macrophages (ratio 1:2.5) for 16 h. Mock‐treated cells were used as a negative control.

### Isolation of PBNs and monocytes

Blood from SA and non-severe asthma (non-SA) patients were layered onto a Lymphoprep™ solution (Axis‐Shield, Oslo, Norway), followed by centrifugation at 2500 rpm at 20 °C for 25 min without braking. The layer containing peripheral blood mononuclear cells was collected; after red blood cells were completely eliminated by hypotonic lysis, monocytes were isolated for further experiments.

The layer containing red blood cells and granulocytes was sedimented in Hank’s balanced salt solution (HBSS) containing 2 mM ethylenediaminetetraacetic acid (EDTA) and 2% dextran at 37 °C for 45 min. The neutrophil‐rich layer was collected, and red blood cells were lysed. Then, PBEs were isolated from the neutrophil‐rich layer according to the manufacturer’s protocol. PBNs were identified as magnetically labeled non-eosinophils and collected by eluting the retained cells. Isolated PBNs were maintained in RPMI‐1640 medium supplemented with 2% heat‐inactivated fetal bovine serum. Monocyte Isolation Kit II, Eosinophil Isolation Kit, and MACS Columns were purchased from Miltenyi Biotec Inc. (Auburn, CA, USA). Cell purity (>95%) was assessed by H&E staining and flow cytometry based on CD68 and CD11b expression in neutrophils, Siglec‐8, and eosinophil cationic protein (ECP) expression in eosinophils, and CD14 expression in monocytes^23^.

### Measurement of S100A9 in PBNs and confocal microscopy

PBNs isolated from asthmatic patients and HCs were untreated or treated with 100 ng/mL LPS for 2 h for ELISA. In some experiments, PBNs were primed with anti-S100A9 antibodies for 30 min and treated with 100 ng/mL recombinant human S100A9 or LPS for the indicated time (for western blot analysis), 6 h (for confocal microscopy analysis) or 24 h (for cytokine measurement by ELISA).

To detect NET formation by using immunofluorescence, 4 × 10^6^ cells were seeded on poly l‐lysine‐coated slides (Polysciences, Warrington, PA, USA) and then treated with or without the indicated reagents for 6 h. The sections were incubated overnight with anti-myeloperoxidase (MPO), anti-S100A9 (Abcam), and anti-neutrophil elastase (NE) antibodies (Santa Cruz Biotechnology, Inc., Dallas, TX, USA), followed by incubation with Alexa Fluor 488-conjugated donkey anti-rabbit and 594-conjugated donkey anti-goat antibodies (ThermoFisher Scientific) for 1 h. The slides were incubated with DAPI (1:1.000) for 5 min. The images were taken with a confocal laser scanning microscope (LSM710, Cal Zeiss Microscopy GmbH, Jena, Germany)

### NET quantification

PBNs were suspended in phenol red-free RPMI with 2% FBS and antibiotics. Cells were placed on ice at least 30 min after isolation to prevent cell clumping and activation, and then 4 × 10^6^ PBNs were seeded in a 24-well plate and stimulated with the targeted drugs as described above. Next, 1 U/mL micrococcal nuclease (ThermoFisher Scientific) was added and incubated for 20 min at 37 °C. The supernatant was used to measure dsDNA with a Quant-IT pico-green dsDNA kit (Invitrogen, Paisley, UK) according to the manufacturer’s instructions. The signal was measured at excitation and emission wavelengths of 480 and 520 nm, respectively, with a fluorescence microplate reader (Synergy HT; BioTek Instrument, Inc., Winooski, VT, USA).

### PBN migration and ROS assays

Isolated PBNs were stained with 2 μmol of calcium aceto-methyl ester (Life Technologies, Eugene, OR, USA) for the migration assay and 2′,7′-dichlorofluorescein diacetate (H2DCFDA) (Life Technologies) for the ROS assay for 30 min at 37 °C, after which the cells were washed once with 1× HBSS. For the migration assay, the cells were pretreated with anti-S100A9 for 30 min and then seeded on the upper chamber with a pore size of 3.0 μm (Neuro Probe, Gaithersburg, MD, USA). Phenol red-free RPMI medium containing S100A9 was added to the lower chamber, and the Transwell plate was incubated for 2 h at 37 °C. For the ROS assay, the cells were pretreated with anti-S100A9 for 30 min in phenol red-free RPMI and then treated with S100A9 for 30 min. The signal was measured at excitation and emission wavelengths of 480 and 520 nm, respectively, with a fluorescence microplate reader (Synergy HT; BioTek Instrument).

### Macrophage polarization and activation protocols

Monocytes were isolated and differentiated into macrophages as previously reported^24^. Briefly, monocytes (1 × 10^6^ cells/mL) were seeded on a 6-well plate containing serum-free RPMI 1640 supplemented with 2 mM l-glutamine, 100 U/mL penicillin, and 100 μg/mL streptomycin. After 2 h, nonadherent cells were removed by repeated washing, and the remaining adherent fraction was cultured for 7 days at 37 °C and 5% CO_2_ in the presence of 10% FBS. The medium was not replaced throughout the culture period, and no further exogenous agents were added to induce monocyte polarization into resting macrophages. Next, S100A9 was used to treat M0 macrophages for 48 h for the ICC assay, for the indicated times (3, 6, 12, 24, or 48 h) for western blotting, and for 72 h for ELISA. The supernatant was collected and stored at −20 °C for cytokine measurement.

To compare the level of S100A9 released from macrophages, M0 macrophages were obtained from HCs and asthmatic patients and induced to undergo either M1 polarization with LPS (1 μg/mL) and IFN-γ (10 ng/mL, R&D, Inc., Minneapolis, MN, USA) or M2 polarization with IL-4 (20 ng/mL, R&D) and IL-13 (5 ng/mL, R&D) for 72 h. In cocultures of A549 cells and macrophages, M0 macrophages were seeded in the upper chamber of a Transwell system (Neuro Probe) with LPS and IFN-γ for 72 h to induce M1 polarization. The cells were washed with 1× HBSS three times and cocultured with A549 cells. LPS was added to the lower chamber and incubated for 24 h.

### ELISA

Human S100A9, IL-6, TNF-α, IL-1β, IFN‐γ, and IL‐8 were measured using kits (R&D Systems Inc., Minneapolis, MN, USA) according to the manufacturer’s instructions. For mouse BALF and serum, S100A9, IL-5, IL-13, keratinocytes-derived chemokine (KC), and interferon-gamma (IFN-γ) were measured using ELISA kits (R&D Systems) according to the manufacturer’s instructions.

### Western blotting

Cells were lysed with lysis buffer containing a protease/phosphatase inhibitor cocktail (Cell Signaling Technology, Danvers, MA, USA). We used anti-S100A9 (Abcam for humans and R&D for mice), anti-phospho-S100A9 (ThermoFisher Scientific), anti-phospho-ERK (Abcam), anti-ERK (Abcam), anti-phospho-p38 (Abcam), anti-p38 (Abcam), anti-phospho-NF-κB (Abcam), anti-NF-κB (Cell Signaling Technology), anti-phospho‐Smad 3 (Abcam), anti-Smad 3 (Abcam), anti-MPO (Abcam), anti-NE (Santa Cruz Biotechnology), anti-CD68 (Abcam), anti-inducible nitric oxide synthase (iNOS) (Abcam), and anti-Arginase 1 (ThermoFisher Scientific) antibodies.

### In vivo experiments

Six‐week‐old female BALB/c mice (Jackson Laboratory, Bar Harbor, ME, USA) were used under specific pathogen‐free conditions. All experimental protocols were approved by the Institutional Animal Care and Use Committee of Ajou University (IACUC 2018-0041). To compare the level of S100A9 among the EA, NA, and MA phenotypes, we established different asthma mouse models according to a previous protocol^25^. On days 0 and 7, the mice were intraperitoneally sensitized with 10 µg of ovalbumin (OVA, Sigma‐Aldrich) in aluminum hydroxide (Alum, Sigma‐Aldrich) solution. From days 14 to 17, the mice were challenged with 6% OVA for 30 min using an ultrasonic nebulizer (NE-SM1; Ktmed Inc., Seoul, South Korea). To establish the mouse models of NA and MA, mice were intranasally administered LPS. The MA group received 1 µg of LPS on day 15, while the NA group received 10 µg of LPS from days 15 to 17. Then, the mice were challenged with 6% OVA for 30 min using an ultrasonic nebulizer 30 min after LPS administration. The mice were examined 24 h after the last challenge^25^. Airway resistance to inhaled MCh (Sigma‐Aldrich) was measured using the FlexiVent System (SCIREQ, Montreal, Canada) as previously described^26^. Lungs were fixed in 4% formalin, embedded in paraffin, and cut into 5‐μm sections. H&E staining was conducted to investigate immune cell infiltration. PAS staining was performed to determine mucus production in the lungs. Slides were stained with anti-S100A9 and anti-MPO to determine the expression of S100A9 in neutrophils in lung tissues.

### Statistical analysis

Differences between the two groups were analyzed by Student’s *t*-test or the Mann–Whitney *U*‐test for continuous variables and Pearson’s chi-squared test for categorical variables. Comparisons of data from multiple groups were made by using one-way ANOVA with Bonferroni’s post hoc test. Receiver operating characteristic (ROC) curve analysis was performed to estimate the diagnostic value of serum cytokines. Statistical analyses were performed using SPSS software version 22.0 (IBM Corp., Armonk, NY, USA). GraphPad Prism 6.0 software (GraphPad Inc., San Diego, CA, USA) was used to produce the graphs.

## Results

### Clinical characteristics of the study subjects

**Fig. 1:**
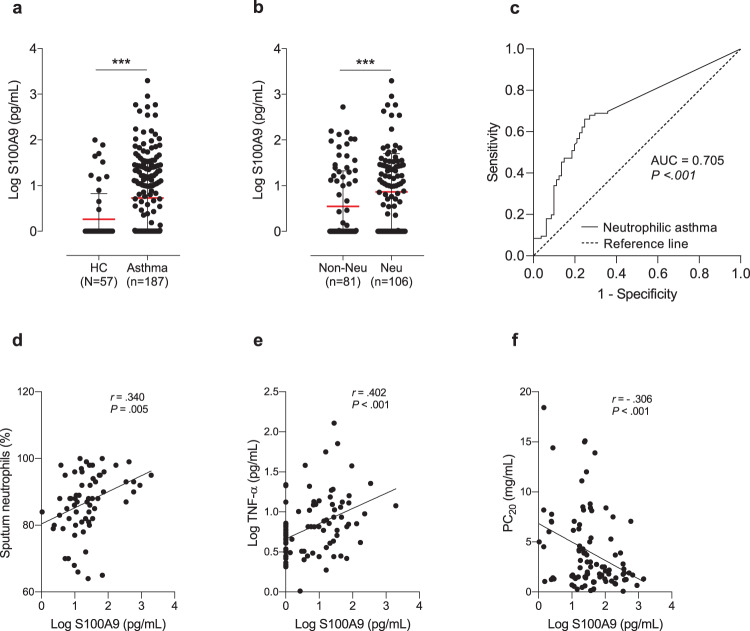
Clinical implications of serum S100A9 in neutrophilic asthma (NA). Comparisons of serum S100A9 levels between **a** HCs and asthmatic patients and between **b** patients with non-NA and NA. The data are presented as the means ± SD. *P* values were obtained by *P < 0.050, **P < 0.010, ***P < 0.001 were obtained by Student’s *t*-test. **c** The receiver operating characteristic curve of serum S100A9 levels for discriminating NA from non-NA asthma. Correlations between serum S100A9 levels and **d** sputum neutrophil counts/**e** serum TNF-α levels/**f** PC_20_ methacholine values. The data are presented as the Pearson correlation coefficient *r* (*P* value). AUC, area under the curve; FEV_1_%, forced expiratory volume in 1 s; HC, healthy control; PC_20_, the concentration of methacholine required to cause a 20% decrease in FEV_1_; ROC, receiver operating characteristic; S100A9, S100 calcium-binding protein A9; TNF-α, tumor necrosis factor-α.

Supplementary Table S1 shows the demographic characteristics of the subjects. Asthmatic patients had a higher incidence of atopy than HCs (*P* = 0.001), and 22.2% had SA. Patients with NA had reduced MCh-PC_20_ values, peripheral eosinophil counts, and sputum eosinophil counts, as well as increased sputum neutrophil counts (*P* < 0.050 for all). Serum S100A9 levels (log-transformed values) were significantly higher in asthmatic patients than in HCs (0.7 ± 0.8 vs 0.2 ± 0.6 pg/mL, *P* < 0.001; Fig. 1a), and significantly higher levels were noted in patients with NA than in those without NA (0.9 ± 0.8 vs 0.5 ± 0.8 pg/mL, *P* = 0.001; Fig. 1b). Serum S100A9 level could discriminate the NA group from the non-NA group at the optimal cutoff value of 5.448 pg/mL, with 60.4% sensitivity and 76.5% specificity (AUC = 0.705, 95% CI = 0.629–0.780, *P* < 0.001) (Fig. 1c). In patients with NA but not non-NA patients, positive correlations were found between serum S100A9 and sputum neutrophil counts/serum TNF-α (*r* = 0.340, *P* = 0.005; *r* = 0.402, *P* < 0.001, respectively) (Fig. 1d, e) as well as serum IL-8 (*r* = 0.201, *P* = 0.046)/MPO (*r* = 0.264, *P* = 0.017) (Supplementary Fig. S1), and a negative correlation between S100A9 and MCh-PC_20_ values was noted (*r* = −0.306, *P* < 0.001; Fig. 1f)_._

Asthmatic patients were classified into S100A9-high and S100A9-low groups (the cutoff value based on the mean + 2 SD of the HC group was 43.904 pg/mL), and their clinical parameters and serum cytokine levels were compared between the 2 groups. The S100A9‐high group had a significantly higher incidence of SA (46.2% vs 19.9%, *P* = 0.006), lower MCh-PC_20_ values (3.2 ± 4.6 vs 5.2 ± 6.1 mg/mL, *P* = 0.032), and higher levels of serum IL-6/IL-17/TNF-α (*P* = 0.040, *P* = 0.019, and *P* = 0.024, respectively) as well as higher sputum neutrophil counts (71.5 ± 35.3% vs 57.5 ± 34.5%, *P* = 0.008; Supplementary Table S2).

### Effects of LPS on airway inflammation and S100A9 production by AECs

**Fig. 2:**
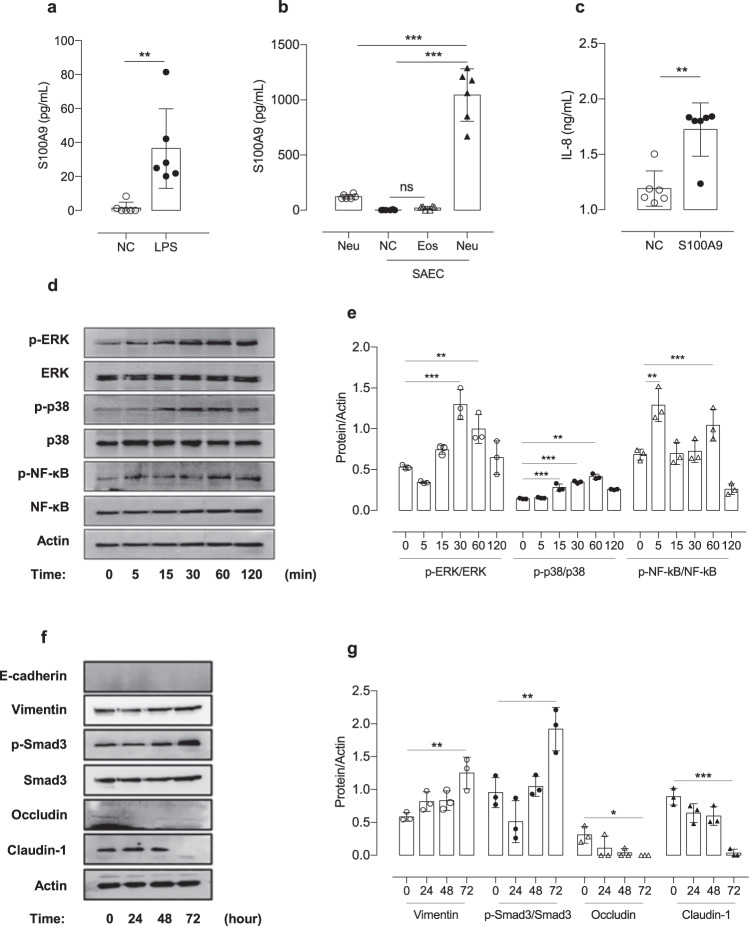
Effects of LPS-induced S100A9 on airway epithelial cell stimulation. The concentrations of S100A9 released from SAECs **a** treated with LPS or **b** cocultured with human peripheral granulocytes. **c** The effects of S100A9 on the production of IL-8 in SAECs. **d** The time-dependent phosphorylation of ERK, p38, and NF-κB in cells stimulated with S100A9. **f** Time-dependent changes in tight junction protein levels and the phosphorylation of Smad 3 in SAECs stimulated by S100A9. **e**, **g** Representative intracellular expression data in at least three independent experiments. The data are presented as the means ± SD. *P < 0.050, **P < 0.010, ***P < 0.001 were obtained by one‐way ANOVA with Bonferroni’s post hoc test. ERK extracellular signal-regulated kinase, Eos eosinophils, LPS lipopolysaccharide, NF-κB nuclear factor kappa B, Neu neutrophils, S100A9 S100 calcium-binding protein A9, SAEC small airway epithelial cell.

The mechanisms by which S100A9 is produced by A549 cells and SAECs in NA were investigated in vitro using LPS. When A549 cells and SAECs were cultured and incubated with LPS for 24 h, significantly increased production of S100A9 was noted in both cell lines (*P* < 0.001 for A549 cells, *P* = 0.004 for SAECs), as shown in Fig. 2a and Supplementary Fig. S2a. In addition, when A549 cells and SAECs were cocultured with PBNs obtained from asthmatic patients for 16 h, S100A9 production was further increased (*P* < 0.001 for both) in PBN-stimulated cells but not in PBE-stimulated cells (Fig. 2b and Supplementary Fig. S2b).

When S100A9 was incubated with A549 cells and SAECs, S100A9-induced IL-8 production via phosphorylation-mediated p38, ERK, and NF-κB pathway activation in a time-dependent manner (*P* < 0.050; Fig. 2c–e and Supplementary Fig. S2c–e). Additionally, S100A9-treated AECs exhibited downregulated expression of E-cadherin and claudin-1 and enhanced expression of phosphorylated Smad 3 in both A549 cells and SAECs (Fig. 2f, g and Supplementary Fig. S2f, g).

### Effects of S100A9 on neutrophil activation and NET formation

**Fig. 3:**
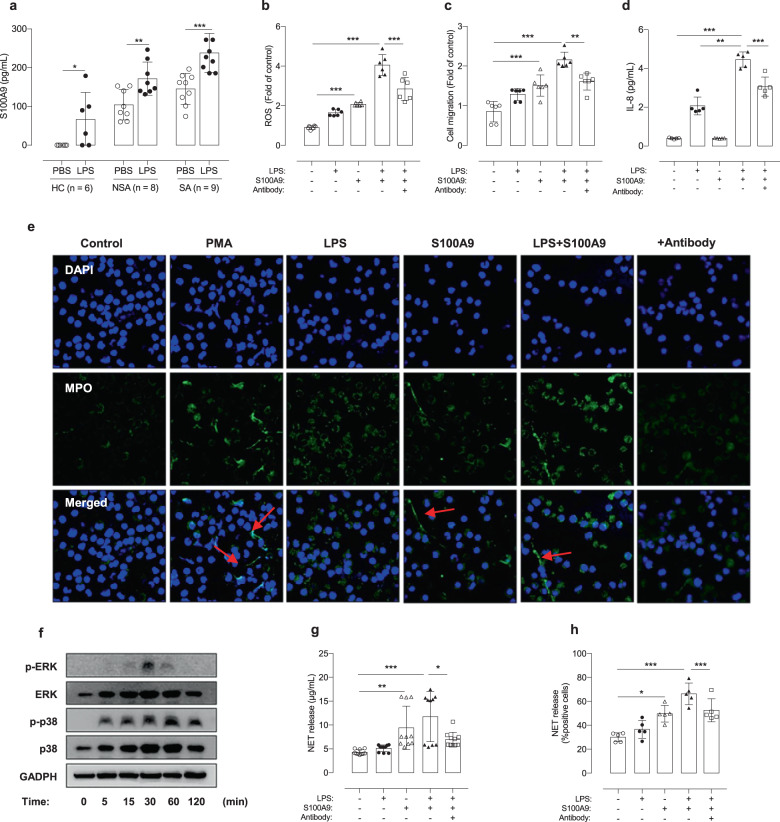
Effects of LPS on S100A9-induced release of extracellular traps from human peripheral neutrophils (PBNs). **a** Effects of LPS on the induction of S100A9 in PBNs. **b** ROS formation in PBNs under LPS/S100A9 stimulation. **c** The percentage of migrating PBNs under LPS/S100A9 stimulation. **d** The levels of IL-8 produced by PBNs under various conditions. **e** The formation of NETs as observed by using confocal microscopy. Scale bar, 25 μm. **f** The time-dependent phosphorylation of ERK and p38 in PBNs treated with S100A9. **g** NET levels were evaluated by extracellular DNA concentrations were measured using the PicoGreen assay and **h** using confocal microscopy. The data are presented as the means ± SD. *P < 0.050, **P < 0.010, ***P < 0.001 were obtained by one‐way ANOVA with Bonferroni’s post hoc test. DAPI 4′,6-diamidino-2-phenylindole, ERK extracellular signal-regulated kinase, HC healthy control, IL interleukin, LPS lipopolysaccharide, MPO myeloperoxidase, NE neutrophil elastase, NET neutrophil extracellular trap, NSA non-severe asthma, PMA phorbol 12-myristate 13-acetate, S100A9 S100 calcium-binding protein A9, SA severe asthma.

PBNs were isolated from non-SA and SA patients, as well as HCs. After stimulation with LPS, significantly greater production of S100A9 (baseline and LPS-stimulated levels) was observed in asthmatic patients than in HCs; among asthmatic patients, significantly greater production of S100A9 was noted in patients with SA than in non-SA patients (*P* < 0.050 for non-SA and *P* < 0.001 for SA; Fig. 3a). Additionally, recombinant S100A9-induced ROS production in PBNs, as measured by the H2DCFDA assay, and recruited PBNs were evaluated by Transwell migration assays (*P* < 0.001 for both; Fig. 3b, c). Anti-S100A9 treatment suppressed PMA-induced NET formation, ROS production, and neutrophil migration (*P* < 0.050 for all; Supplementary Fig. S3). Although S100A9 did not induce cytokine production by PBNs, S100A9-primed PBNs exhibited significantly enhanced LPS-induced production of cytokines, including IL-8, and this effect was inhibited by anti-S100A9 treatment (*P* < 0.001; Fig. 3d) but not by dexamethasone treatment (data not shown).

After activation and migration, S100A9-primed PBNs exhibited increased expression of phospho-p38 and phospho-ERK in a time-dependent manner, which was evaluated by western blotting. Consequently, NET formation was observed by ICC assays in asthmatic patients (Fig. 3e, f) but not in HCs (data not shown). Quantification of NET levels by using the PicoGreen assay showed that S100A9‐treated PBNs from asthmatic patients produced significantly higher levels of extracellular DNA (ecDNA) than in HCs and the LPS-treated group (*P* < 0.010, *P* = 0.034; Fig. 3g). However, the anti-S100A9 antibody but not dexamethasone suppressed the level of ecDNA (*P* = 0.013; Fig. 3h).

### Effects of S100A9 on macrophage polarization and proinflammatory cytokine release

**Fig. 4:**
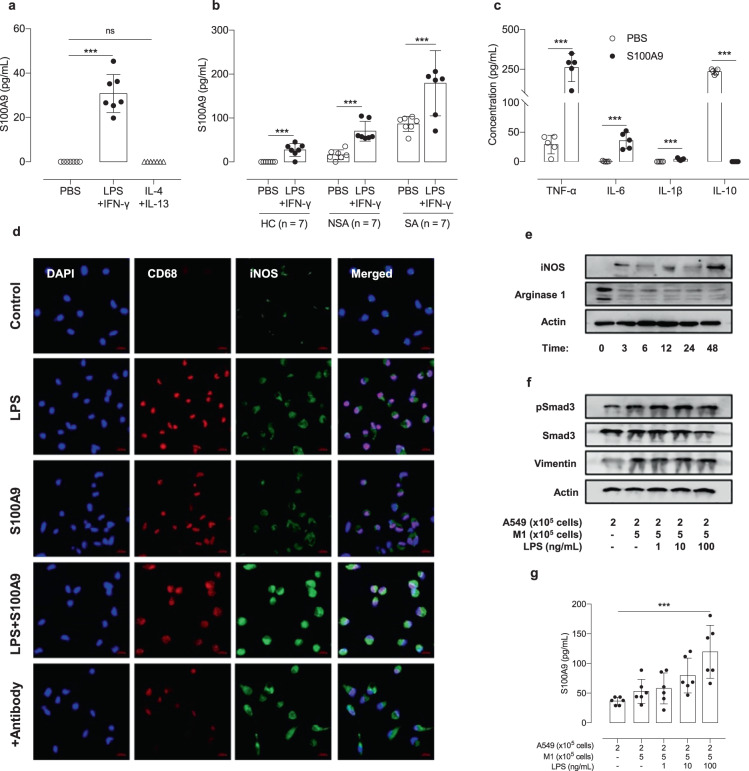
Effects of S100A9 on macrophage activation and polarization. **a** The enhanced production of S100A9 induced by various stimuli was evaluated by ELISA. **b** The effects of LPS and IFN-γ on the production of S100A9 by M0 macrophages were evaluated by ELISA. **c** The concentrations of proinflammatory cytokines released from M0 macrophages after S100A9 stimulation. **d** Immunofluorescent staining for CD68 (red) and iNOS (green) in M0 macrophages. Scale bar, 25 μm. **e** The expression of S100A9-induced iNOS and Arginase 1 was quantified by western blotting. **f** Enhanced Smad 3 signaling in A549 cells induced by M1 macrophages. **g** The LPS-induced S100A9 release from A549 cells was augmented by M1 macrophages. The data are presented as the means ± SD. *P < 0.050, **P < 0.010, ***P < 0.001 were obtained by one‐way ANOVA with Bonferroni’s post hoc test. HC healthy control, iNOS inducible nitric oxide synthase, IFN-γ interferon-gamma, IL interleukin, LPS lipopolysaccharide, M1 M1 macrophage, NSA non-severe asthma, S100A9 S100 calcium-binding protein A9, SA severe asthma, TNF-α tumor necrosis factor-α.

M0 macrophages produced S100A9 in response to LPS and IFN-γ (not to IL-4 and IL-13), and these levels were significantly higher in M0 macrophages from SA patients than in those from non-SA patients or HCs (*P* < 0.001 both; Fig. 4a, b). M0 macrophages were stimulated by S100A9 and then polarized into M1 cells, and there was an increase in M1 cytokines, including TNF-α, IL-6, and IL-1β (*P* < 0.001), and a decrease in M2 cytokines, including IL-10 (*P* < 0.001) (Fig. 4c).

Then, S100A9 or the anti-S100A9 antibody was used to stimulate M0 macrophages, and S100A9 increased the expression of CD68 (the marker for macrophage maturation) and iNOS (M1 markers), while these effects were attenuated by anti-S100A9 antibody treatment (Fig. 4d) but not by dexamethasone (data not shown). The expression of iNOS was increased by S100A9 stimulation in a time-dependent manner, while the expression of Arginase 1 was decreased in M0 macrophages (Fig. 4e). When A549 cells were cocultured with M1 macrophages (with or without LPS‐pretreated cells), they changed in shape (elongated form, data not shown). Moreover, the expression of vimentin and phosphorylated Smad 3 was enhanced by LPS stimulation (Fig. 4f). Furthermore, S100A9 levels were significantly increased in A549 cells cocultured with M1 macrophages after stimulation with 100 ng/mL LPS (*P* < 0.001) (Fig. 4g).

### Increased levels of S100A9 in sera/BALF in the mouse model of NA

**Fig. 5:**
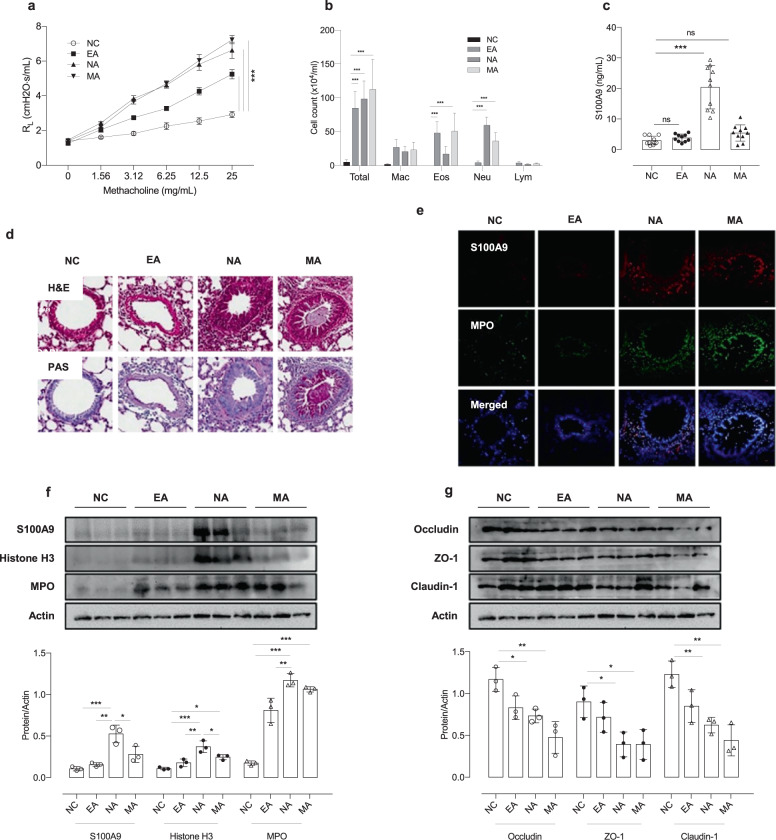
LPS-induced S100A9 and neutrophil activation in vivo. **a** Airway hyperresponsiveness. **b** Differential cell counts. **c** Levels of S100A9 in BALF. **d** Lung tissues stained with H&E (upper panel) or PAS (lower panel). Scale bar, 200 μm. **e** Immunofluorescent staining for S100A9 (red) and myeloperoxidase (green) in lung tissues. Scale bar, 25 µm. **f** The expression of S100A9, histone H3 and MPO in lung tissues. **g** Changes in the expression of tight junction proteins in lung tissues. The data are presented as the means ± SD. *P < 0.050, **P < 0.010, ***P < 0.001 were obtained by one‐way ANOVA with Bonferroni’s post hoc test. EA eosinophilic asthma, Eos eosinophil, IL interleukin, LPS lipopolysaccharide, Lym lymphocytes, MA mixed granulocytic asthma, Mac macrophage, MPO myeloperoxidase, NA neutrophilic asthma, NC normal control, NE neutrophil elastase, Neu neutrophil, S100A9, S100 calcium-binding protein A9.

The role of S100A9 in NA was confirmed in vivo using 3 types of murine asthma models (NA, EA, and MA). AHR was significantly increased in the NA and MA groups, as well as the EA group, compared to NC group (*P* < 0.001 for all) (Fig. 5a). Significantly increased numbers of total cells, neutrophil counts, and S100A9 levels in BALF were noted in the NA and MA groups compared to the EA group, and the highest neutrophil counts and S100A9 levels were noted in the NA group compared to the other 2 asthma groups (*P* < 0.001 for all) (Fig. 5b, c). IFN-γ, IL-13, IL-17A, and KC were significantly higher in NA and MA groups compared to NC groups (*P* < 0.001; Supplementary Fig. S4). An increased number of double-positive cells (S100A9 plus MPO and S100A9 plus iNOS) infiltrated bronchial epithelial cells and the peribronchial area in the NA group compared to the other 2 groups (Fig. 5d, e and Supplementary Fig. S5). Additionally, enhanced expression of histone H3, S100A9 and MPO was noted in the lung homogenates of the NA group (Fig. 5f). In contrast, the expression of tight junction proteins, including occludin, claudin-1, and ZO-1, was decreased in the NA group (Fig. 5g).

## Discussion

S100A9, which reflects neutrophil activation status (rather than S100A8), has been demonstrated to play a role in the pathogenic mechanisms of NA; however, the exact mechanism is still unclear^15–18,27^. The present study investigated the role of S100A9 in neutrophil airway inflammation in the context of the interactions between neutrophils and AECs/macrophages in patients with NA. Significantly higher levels of S100A9 were found in the sera of adult asthmatic patients than in HCs, as well as in the NA group relative to the non-NA group, and there was a significant correlation between the S100A9 level and sputum neutrophil counts. Patients with SA had higher serum S100A9 levels than those with non-SA. In addition, the serum S100A9 level could discriminate NA patients from non-NA patients and HCs, suggesting that serum S100A9 is a potential biomarker for predicting the phenotype of NA. In vitro experiments demonstrated that LPS exposure could stimulate S100A9 production by AECs, PBNs, and M0 macrophages, further enhancing the activation of AECs and IL-8 production, followed by immune cell activation, including that of neutrophils^23^. Moreover, S100A9 could induce NET formation in PBNs (which enhances epithelial dysfunction and type 2 inflammation) and M1 macrophage polarization, all of which were attenuated by anti-S100A9 treatment. These findings suggest that S100A9 plays a critical role in maintaining and perpetuating neutrophilic airway inflammation in NA, contributing to the progression to SA.

Neutrophilia in asthma has been reported to be associated with bacterial endotoxin and air pollution exposure^28,29^. Among them, endotoxin exposure (bacterial infection) is a trigger for neutrophilic inflammation and asthma exacerbation^30^. Therefore, LPS has been used to stimulate AECs, neutrophils, and macrophages in vivo and in vitro. Regarding the relationship between neutrophils and airway dysfunction in asthma, NA has been shown to be associated with reduced pre-post-bronchodilator FEV_1_% in the United Kingdom (UK) population^31^. In patients with NA, sputum S100A9 levels were significantly higher in those with an uncontrolled status than in those with a well-controlled status, suggesting associations with poor airway function and clinical outcomes^17^. The present study showed that NA patients had significantly lower MCh-PC_20_ levels and higher S100A9 levels than non-NA patients, and there was a negative correlation between S100A9 and MCh-PC_20_ levels; among asthmatic patients, the high-S100A9 group had lower MCh-PC_20_ levels than the low-S100A9 group. Moreover, our in vivo model of NA showed significantly higher S100A9 levels and lower AHR than in EA. In vitro studies demonstrated that when exposed to PBNs, AECs increased S100A9 production, leading to AEC/neutrophil activation and NET formation, which further enhanced AEC activation and dysfunction (with increased expression of vimentin and Smad 3 signaling). These findings suggest that S100A9 may be a key mediator that drives neutrophilic airway inflammation and remodeling via interactions between AECs and neutrophils in asthma (the AEC-S100A9–neutrophil axis), ultimately contributing to poor clinical outcomes. Taken together, these findings suggest that activated neutrophils with increased S100A9 may exacerbate AHR, airway inflammation, and remodeling in both neutrophilic and type 2 asthmatic patients. New therapeutic interventions are needed to control this axis.

MPO and NE, two major components of NETs, are released from activated neutrophils. MPO can induce hypochlorous acid production, which causes resident cell damage in the lungs^32^. Greater MPO production was noted in neutrophils from SA patients than in neutrophils from non-SA patients or HCs^33,34^. A recent study provided evidence for the role of NETs in SA; increased levels of NETs in SA could enhance type 2 airway inflammation by increasing eosinophil degranulation and AEC dysfunction^23^. In addition, NETs could increase autoantigen production by AECs, contributing to asthma severity, as validated in an animal model of asthma^8,35^. The present study demonstrated that S100A9 could induce NET formation in PBNs from asthmatic patients. Greater production of S100A9 was noted in patients with SA than in those without SA. In addition, S100A9-induced NET formation was mediated through the p38 and ERK pathways (higher in SA than in non-SA), and this effect could be suppressed by anti-S100A9 treatment. A previous report demonstrated that NETs could enhance type 2 inflammation and epithelial dysfunction, indicating that S100A9 could contribute to the severity of type 2 inflammation, as well as neutrophilic inflammation, via NET formation^23^. Taken together, these findings suggest that S100A9 can contribute to persistent neutrophilic inflammation, as well as type 2 inflammation, which may be a potential therapeutic target, especially in NA.

We evaluated the role of S100A9 in macrophage activation because (1) macrophages produce S100A9 during chronic inflammation and (2) increased expression of S100A9 was found in peripheral blood mononuclear cells from patients with atherothrombosis^36,37^. Macrophage polarization contributes to airway inflammation and remodeling in asthma by facilitating phagocytosis, efferocytosis, and inflammatory cytokine release rather than changes in cell number^38^. M1 macrophages play a key role in initiating and regulating proinflammatory cytokine production, leading to the recruitment of neutrophils, while M2 macrophages have an immunosuppressive role and attenuate airway inflammation^38,39^. Regarding the interactions between S100A9 and macrophages, the present study demonstrated that patients with higher S100A9 levels had higher serum IL-6 and TNF-α levels than those with low-S100A9 levels, and there was a positive correlation between serum S100A9 and serum TNF-α in patients with NA (not in those with EA). Increased levels of IL-6 released by macrophages and AECs in sputa from asthmatic patients were reported and had a negative correlation with the peak expiratory flow rate (PEFR)^40^. Additionally, in vivo experiments indicated that macrophage-derived IL-6 promotes house dust mite-induced airway inflammation^41^. In addition, increased levels of TNF-α (from macrophages and mast cells) were found in sputum samples from asthmatic patients and were associated with sputum neutrophil counts and disease severity^42,43^. The present study demonstrated significantly higher production of S100A9 (spontaneous and LPS-induced responses) by macrophages from asthmatic patients than those from HCs; among asthmatic patients, significantly higher S100A9 production was noted in macrophages from patients with SA than in those from non-SA patients. S100A9 could results in the production of proinflammatory and remodeling cytokines, including IL-6, IL-1β, and TNF-α, by macrophages and facilitate LPS-induced macrophage polarization, and these effects were suppressed by anti-S100A9 treatment. In addition, PBNs obtained from patients with severe asthma were found to induce increased production of IL-1β, IL-6, and IL-8 in response to LPS plus S100A9, which further activated macrophages and AECs. These findings suggest that S100A9 may be a key mediator that polarizes M1 macrophages by activating neutrophils, facilitating proinflammatory cytokine production, shifting to non-type 2 airway inflammation/remodeling, and progressing to SA; S100A9 may be a potential therapeutic target for suppressing M1-mediated neutrophilic inflammation via the AEC-S100A9-macrophage polarization–neutrophil axis in NA.

This study has two limitations. One is that it is a single-center study; further replication studies with larger sample sizes are needed to confirm our results. The other is that functional differences in S100A9 need to be clarified between the NA and MA groups. Despite these limitations, we first demonstrated the comprehensive role of S100A9 in airway inflammatory mechanisms in NA in 2 ways: (1) the AEC-S100A9–neutrophil axis and (2) AEC-S100A9-macrophage polarization.

In conclusion, the results of this study suggest that S100A9 may play a pivotal role in NA by enhancing neutrophil activation, NET formation, and macrophage polarization, and S100A9 is a therapeutic target of neutrophilic airway inflammation, especially in patients with SA.

## Supplementary information

Supplemental tables and figures
