# Author Correction: S100A9 in adult asthmatic patients: a biomarker for neutrophilic asthma

**DOI:** 10.1038/s12276-022-00826-9

**Published:** 2022-08-09

**Authors:** Quang Luu Quoc, Youngwoo Choi, Tra Cao Thi Bich, Eun-Mi Yang, Yoo Seob Shin, Hae-Sim Park

**Affiliations:** 1 Department of Allergy and Clinical Immunology, Ajou University School of Medicine, Suwon, South Korea; 2 Department of Biomedical Sciences, Ajou University School of Medicine, Suwon, South Korea

**Keywords:** Prognostic markers, Adaptive immunity, Immunological disorders, Diagnostic markers

Correction to: *Experimental & Molecular Medicine* 10.1038/s12276-021-00652-5, published online 20 July 2021

After online publication of this article, the authors noticed an error in the Supplementary figure (Figure S3a) section.

The correct statement of this article should have read as below.

“The authors report an error in Figure S3a in the Supplementary figure section. The authors regret that Figure S3a of this paper has an error in image arrangement in the confocal of S100A9 and NE expressions in peripheral blood neutrophils with or without anti-S100A9 treatment. The updated figure is provided below. This did not impact the analysis, results, or conclusions of the article.”

The authors apologize for any inconvenience caused.

The original article has been corrected.

## Supplementary information

Figure S3a

