# Supplementary material for: S100A9 in adult asthmatic patients: a biomarker for neutrophilic asthma
Source: Exp Mol Med. 2021 Jul 20;53(7):1170–9. doi: 10.1038/s12276-021-00652-5 (PMC8333352; doi:10.1038/s12276-021-00652-5)
Supplement: Supplementary file 1 — Supplemental tables and figures [file 12276_2021_652_MOESM1_ESM.pdf]

SUPPLEMENTARY TABLES

Table S1. Demographic data of the study subjects. Values are given as n (%) for categorical variables and as mean ± SD for continuous variables.

Variables	HC (n = 57)	Asthma (n = 187)	*P* value	Neu (n=106)	Non-Neu (n=81)	*P* value
Age (y)	41.7 ± 14.3	45.8± 14.2	.158	46.4 ± 14.6	45.1 ± 13.6	.705
Female sex (%)	68.4	63.6	.508	69.8	55.6	.032
Atopy (%)	16.7	49.7	.001	55.3	50.8	.599
Severe asthma (%)	NA	22.2	NA	23.6	23.5	.563
Baseline FEV_1 (%)	NA	86.9 ± 20.3	NA	85.5 ± 19.6	91.2 ± 19.6	.254
PC_{20} (mg/mL)	NA	3.9 ± 4.2	NA	3.6 ± 4.1	5.9 ± 6.9	.044
Total IgE (kU/L)	NA	473.5 ± 785.4	NA	435.4 ± 788.0	469.8 ± 669.3	.200
TEC (cells/µL)	NA	534.9 ± 936.2	NA	422.5 ± 870.8	541.1 ± 692.1	.021
Sputum eosinophil count (%)	NA	25.5 ± 35.2	NA	5.9 ± 14.5	52.0 ± 37.7	.001
Sputum neutrophil count (%)	NA	58.4 ± 34.7	NA	87.3 ± 8.7	23.0 ± 18.7	.001

P values were given by Pearson chi-square test for categorical variables and Student's t test for continuous variables. FEV_1, forced expiratory volume in 1s; HC, healthy controls; IgE, immunoglobulin E; NA, not applicable; Neu, neutrophilic asthma; Non-Neu, non-neutrophilic asthma; PC_{20}, the provocative concentration of methacholine required to cause a 20% fall in FEV_1; TEC, total eosinophil count.

Table S2. Comparison of demographic characteristics between high-S100A9 and low-S100A9 responders (cutoff value, 43.904 pg/mL).

Variables	High-S100A9 responders (n = 26)	Low-S100A9 responders (n = 161)	P value
Age (y)	42.9 ± 14.5	46.3± 14.1	.246
Female sex (%)	76.9	61.5	.095
Atopy (%)	16.7	49.7	.562
Severe asthma (%)	46.2	19.9	.006
Baseline FEV1 (%)	87.0 ± 12.2	88.8± 20.3	.707
PC20 (mg/mL)	3.2 ± 4.6	5.2 ± 6.1	.032
Total IgE (kU/L)	359.3 ± 467.4	468.1 ± 774.6	.947
TEC (cells/μL)	579.5 ± 1062.7	458.0 ± 750.9	.792
Sputum eosinophil count (%)	27.4 ± 37.8	25.6 ± 35.4	.845
Sputum neutrophil count (%)	71.5 ± 35.3	57.5 ± 34.5	.008
Log IL-6 (pg/mL)	0.2 ± 0.1	0.0 ± 0.2	.040
Log IL-17 (pg/mL)	0.3 ± 0.8	0.0 ± 0.2	.019

Log TNF-α (pg/mL)	0.8 ± 0.6	0.5 ± 0.5	.024

Values are given as n (%) for categorical variables and as mean ± SD for continuous variables. P values were given by Pearson chi-square test for categorical variables and Student's t test for continuous variables. FEV1, forced expiratory volume in 1s; IgE, immunoglobulin E; IL, interleukin; PC_{20}, the provocative concentration of methacholine required to cause a 20% fall in FEV_1; S100A9, S100 calcium binding protein A9; TEC, total eosinophil count; TNF-α, tumor necrosis factor-α; y, year.

SUPPLEMENTARY FIGURES

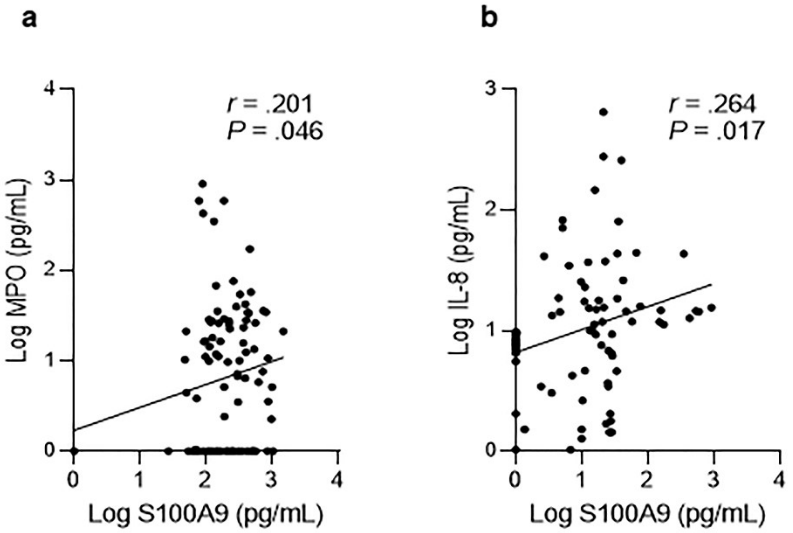

Figure S1. Correlation parameters. The correlations between serum S100A9 and **(a)** serum MPO/ **(b)** serum IL-8 among neutrophilic asthma group. The data are represented as Pearson correlation coefficient r (*P*-value). MPO, myeloperoxidase; S100A9, S100 calcium binding protein A9.

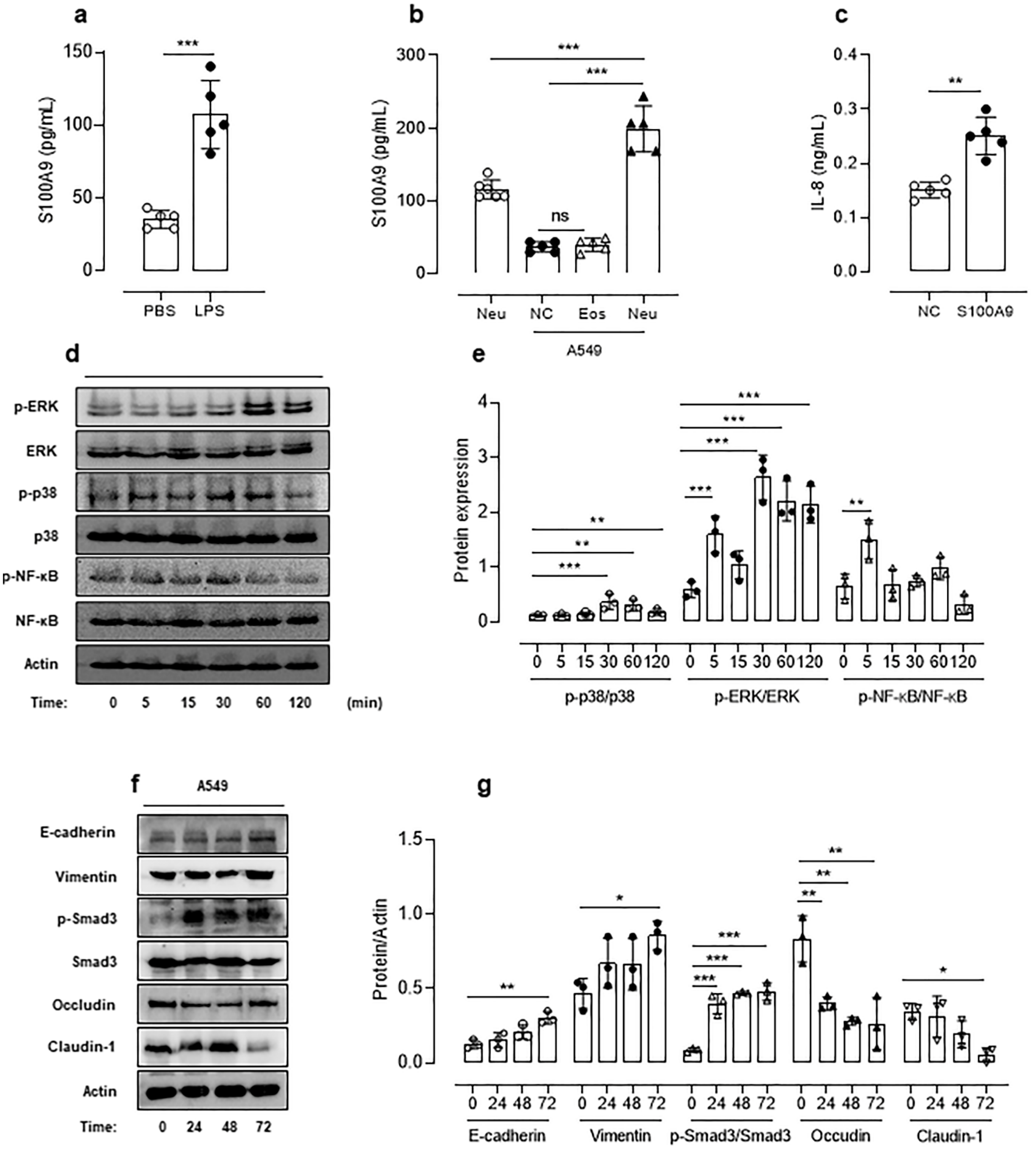

a
S100A9 (pg/mL)
PBS
LPS
b
S100A9 (pg/mL)
ns
Neu
NC
Eos
Neu
A549
c
IL-8 (ng/mL)
NC
S100A9
d
p-ERK
ERK
p-p38
p38
p-NF-κB
NF-κB
Actin
Time: 0 5 15 30 60 120 (min)
e
Protein expression
p-p38/p38
p-ERK/ERK
p-NF-κB/NF-κB
f
A549
E-cadherin
Vimentin
p-Smad3
Smad3
Occludin
Claudin-1
Actin
Time: 0 24 48 72
g
Protein/Actin
E-cadherin
Vimentin
p-Smad3/Smad3
Occudin
Claudin-1

Figure S2. Effects of LPS-induced S100A9 on airway epithelial cell stimulation. Concentrations of S100A9 released from A549 **(a)** treated with LPS or **(b)** cocultured with human peripheral granulocytes. **(c)** Effects of S100A9 on the production of IL-8 in the A549 cells. **(d)** Phosphorylation of ERK, p38, and NF-κB in the cells stimulated with S100A9 in a time-dependent manner. **(f)** Tight-junction proteins and phosphorylation of Smad 3 expression in A549 stimulated by S100A9 in a time-dependent manner. **(e,g)** Representative data of intracellular expressions at least three independent experiments. The data are presented as the means ± SD. $^{*}P < .050$, $^{**}P < .010$, $^{***}P < .001$ were obtained by one-way ANOVA with Bonferroni's post hoc test. ERK, extracellular signal-regulated kinase; Eos, eosinophils; LPS, lipopolysaccharide; NF-κB, nuclear factor kappa B; Neu, neutrophils; S100A9, S100 calcium binding protein A9.

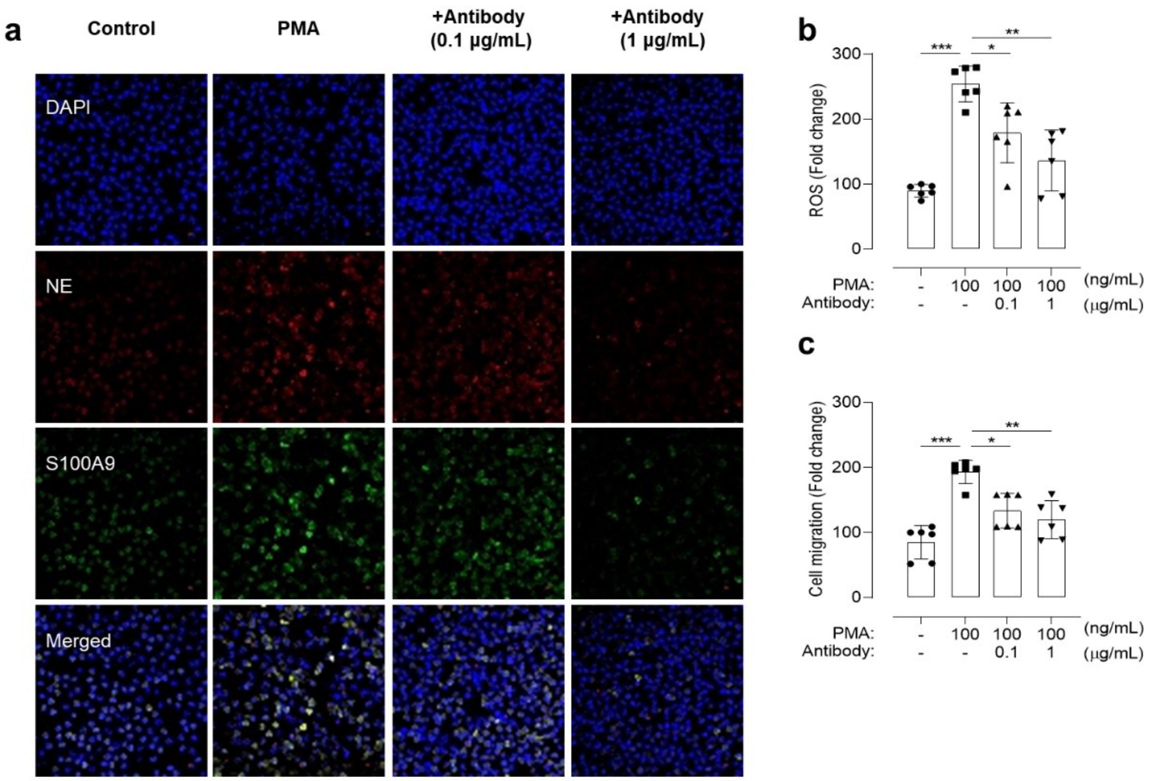

Figure S3. Inhibition of NET formation by anti-S100A9 antibody treatment in human peripheral neutrophils. **(a)** Observation of NETs within S100A9 by using confocal microscopy. Scale bar, 20 μm. **(b)** Evaluation of ROS production in neutrophils. **(c)** Neutrophil migration rate after PMA treatment. Data are presented as means ± SD. n = 6. *P < .05, **P < .01, ***P < .001 were obtained by one-way ANOVA with Bonferroni's post hoc test. DAPI, 4′,6-diamidino-2-phenylindole; NETs, neutrophil extracellular traps; NE, neutrophil elastase; PMA, phorbol 12-myristate 13-acetate; ROS, reactive oxygen species; S100A9, S100 calcium binding protein A9.

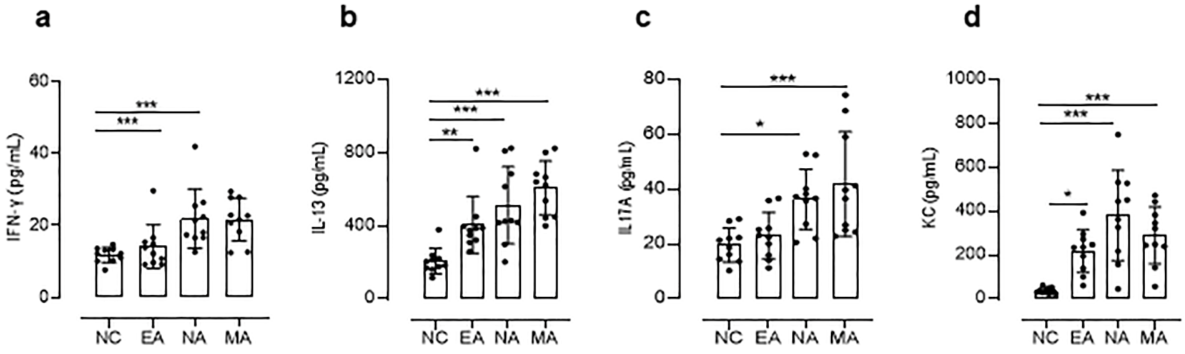

Figure S4. Concentrations of multiple cytokines in BALF. (a) IFN-γ, **(b)** IL13, **(c)** IL17A, and **(d)** KC levels in BALF. Data are presented as means ± SD. *P < .05, **P < .01, and ***P < .001 were obtained by one-way ANOVA with Bonferroni's post hoc test. EA, eosinophilic asthma; IFN-γ, interferon-gamma; KC, keratinocytes-derived chemokine; MA, mixed granulocytic asthma; NA, neutrophilic asthma; NC, normal controls; S100A9, S100 calcium binding protein A9.

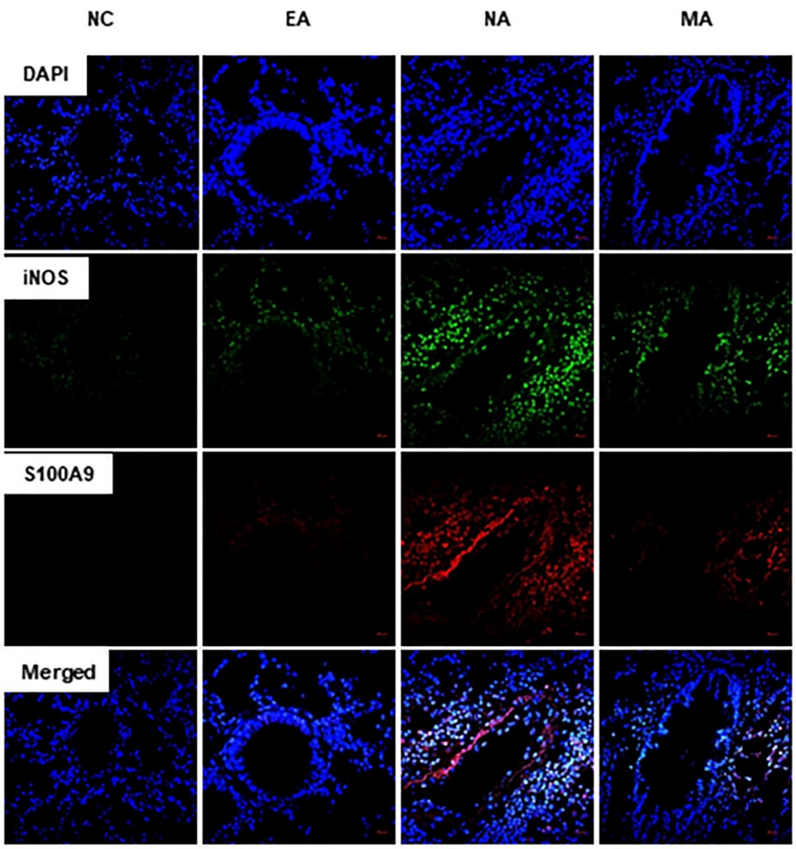

Figure S5. Expression of S100A9 and iNOS in the lung tissues. Scale bar, 25 μm. iNOS, inducible nitric oxide synthase. EA, eosinophilic asthma; MA, mixed granulocytic asthma; NA, neutrophilic asthma; NC, normal controls; S100A9, S100 calcium binding protein A9
