# Supplementary figures and images for: Author Correction: S100A9 in adult asthmatic patients: a biomarker for neutrophilic asthma
Source: Exp Mol Med. 2022 Aug 9;54(8):1308. doi: 10.1038/s12276-022-00826-9 (PMC9440014; doi:10.1038/s12276-022-00826-9)

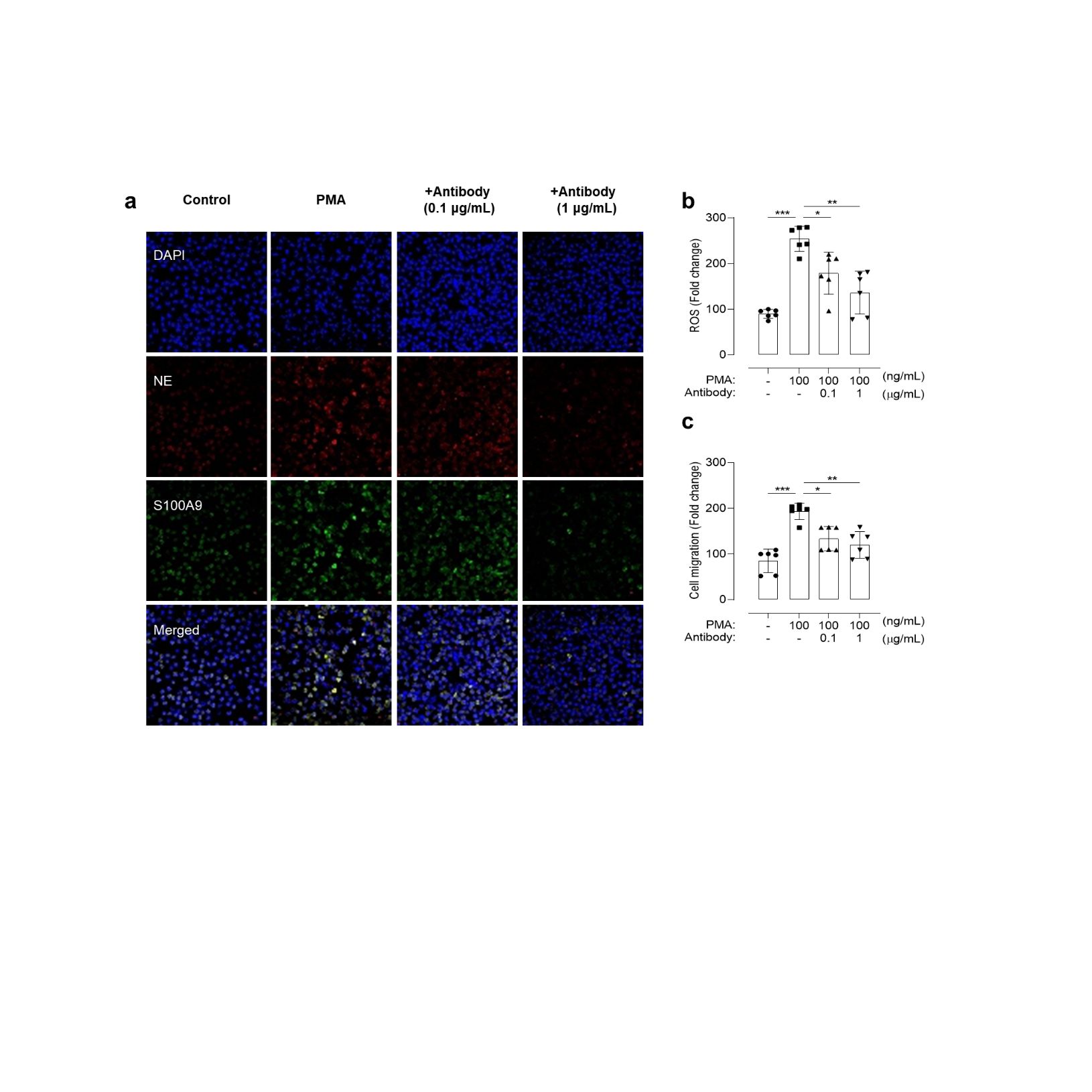

Supplement: Supplementary file 1 — Figure S3a [file 12276_2022_826_MOESM1_ESM.tif]
